# Testing the feasibility of a primary-care exercise intervention to prevent and reverse early frailty and build resilience in community-dwelling older adults

**DOI:** 10.1016/j.eclinm.2022.101355

**Published:** 2022-03-22

**Authors:** John Travers, Roman Romero-Ortuno, Marie-Therese Cooney

**Affiliations:** a School of Medicine, University College Dublin, Dublin; b Trinity College Dublin HSE Specialist Training Programme in General Practice, Dublin; c Global Brain Health Institute, Trinity College Dublin, Dublin; d Mercer's Institute for Successful Aging, St James's Hospital, Dublin; e Department of Geriatric Medicine, St Vincent's University Hospital, Dublin

**Keywords:** Frailty, Resilience, Feasibility, Primary-care, Exercise

## Abstract

**Background:**

Resistance exercises have been shown to prevent and reverse frailty but their application in clinical practice is low. We wished to test the feasibility of an optimised exercise intervention for mild or pre-frailty in a primary-care setting and inform the design of a definitive randomised control trial.

**Methods:**

The intervention was co-designed with eighteen older adults in two group workshops, informed by systematic review and meta-analysis. Eligible patients aged 65+, mildly frail or less, presenting to an Irish primary-care centre over 6 months from January 2020 were invited to participate. They were offered an exercise guide and educational discussion. Demographics, health indicators and frailty scores were recorded. Feasibility was assessed using the Bowen model for acceptability; participation; demand; implementation; practicality; adaptation; integration; expansion; and limited-efficacy. Half of the randomly selected participants were telephoned after one month, and all the participants were called after two to measure effects on adherence.

**Findings:**

94 of 107 eligible people (88%) participated (average age 77, 59 women (63%)). Only 15% had previously considered resistance exercises. The intervention satisfied all Bowen feasibility criteria. At one month, 65% of participants were exercising. At two months, adherence amongst those previously called was higher: 78%. 87% described exercises as ‘very easy’ or ‘somewhat easy’. 66% felt ‘much better’ or ‘slightly better’.

**Interpretation:**

Frailty intervention uptake and adherence were high. A single telephone call appeared to help increase adherence. Participants reported meaningful physical and mental health benefits.

**Funding:**

Roman Romero-Ortuno is funded by a grant from Science Foundation Ireland (SFI), grant number 18/FRL/6188.

**Research in context**

**Evidence before this study**

We searched PubMed, CINAHL, the Cochrane Library register of Controlled Trials and PEDro for English language articles using the terms ("primary care" or "community") and (“screening” or "intervention" or "integrated-care" or “feasibility”) and ("frailty" or "pre-frail"). The search was conducted from inception to May 2018 before study design. Studies on screening, feasibility or interventions aimed at preventing or treating frailty in a primary-care setting were included. There was an absence of feasibility studies of primary-care frailty interventions and participant co-design, despite consensus guidance of the benefits of feasibility assessment and patient involvement before full randomised controlled trials. We identified a heterogeneity of trials, that had not tested initial feasibility, with wide variance of effectiveness.

**Added value of this study**

This is to the best of our knowledge the first feasibility study of a general practitioner (GP)-led exercise intervention to address frailty in the community. The intervention satisfied feasibility criteria in a real-world primary care setting. We observed high intervention uptake and adherence and found that adherence was improved by a single follow up call. Our results may encourage mainstream adoption of feasible primary-care interventions to reverse mild frailty and build resilience in the community.

**Implications of all the available evidence**

The study of frailty is still at an early stage. A broad diversity of frailty interventions have been trialled in recent years, and no definitive approach to tackling this medical syndrome has emerged. Establishing trials on a solid foundation of initial feasibility assessment may contribute to improved acceptability, effectiveness and impact.

Alt-text: Unlabelled box

## Introduction

Frailty has been described as the most problematic expression of population ageing and presents a growing public health challenge.^1^ It is a state of physiological vulnerability to external stressors that increases the risks of illness, falls, dependency, disability, and death.^1^^,^^2^ Population-based longitudinal studies have shown frailty is dynamic, with bidirectional changes between states of ‘non-frailty’, ‘pre-frailty’ and ‘frailty’ possible.^3^^,^^4^ Community prevalence is 11% in over 65-year-olds and 50% in over 80 year-olds and is increasing due to ageing populations.^5^ Resilience is the capacity to recover following exposure to external stressors^6^ and is at the opposing end of a health spectrum to frailty.^7^

A wide range of frailty interventions has been tested in recent years. This reflects an emerging recognition of the opportunity to reverse frailty and its detrimental impact on mortality, morbidity and society, and to build physical resilience. Definitive evidence for effective intervention is lacking, especially for pre-frailty or mild frailty,^8^ though a recent review showed that resistance exercises, along with dietary protein, may be the most effective and easiest to implement interventions to delay or reverse frailty.^9^^,^^10^ However, implementation remains low with only 9% of exercise programmes offered to people with sarcopenia or frailty having resistance exercises as the main focus in a UK study.^11^

There are several reasons why implementation may be so low. Firstly, frailty is a relatively new concept in medical care and research. Only 4 of 46 relevant studies chosen from 925 reviewed papers, pre-dated 2010.^9^ Screening for frailty was mandated in NHS England only in 2017 and international guidelines on frailty screening continue to evolve.^12^ Secondly, many studies have been undertaken in controlled environments that do not reflect a real-life primary-care setting, where multiple, complex and competing demands on resources exist. Thirdly, dissemination of findings from successful intervention trials is just beginning to gather pace and knowledge amongst clinicians about benefits from frailty interventions is in its infancy.

The benefits from successful interventions are significant. Our review showed that 71% of studies measuring impact on frailty status demonstrated significant improvement.^9^ Furthermore, avoiding or reversing frailty can impact mortality, quality of life, and healthcare costs. The risk of death has been shown to be significantly higher, by a factor of almost three, in frail community dwelling adults aged 60 and over.^13^ The annual incremental healthcare cost of frailty per person amongst community dwelling adults has been found to be €12,068 in a German study^14^ and $12,360 (€7940) in a Canadian study.^15^

Community based GPs are in a unique position to offer frailty interventions to older people.^16^ They have typically established long-term, trusting relationships. They are intimately aware of a person's background medical, social, and family history as well as personal preferences. They see people recurrently and can continue to encourage participation in an intervention. This is important for frailty interventions where the benefit depends on continued participation.

Our previous review^9^ and analysis^10^ of interventions enabled us to select the most effective components, while avoiding identified shortcomings, and develop an optimised intervention. However, questions about the feasibility of offering an intervention in primary-care remained.

Feasibility assessment may be indicated when previous interventions had positive outcomes but in different settings to the one of interest^17^; when it is useful to test an intervention in a real-world environment, prioritizing community needs over controlled conditions, to increase generalizability and dissemination^18^; and to determine whether an intervention is appropriate for further efficacy testing in a trial.^17^ Although there have been trials of exercise interventions for frailty in research settings, we could find no feasibility study in the literature that tested a GP led exercise intervention to address frailty in the community.

It seemed appropriate therefore, to assess the feasibility of an optimised intervention to prevent frailty, amongst those who are non-frail or pre-frail, as well as reverse mild frailty and build resilience, in a real-world primary-care setting and establish a foundation for a future definitive randomised control trial (RCT).^19^

## Methods

### Study design

This was a feasibility study set in primary-care practice in Ireland.

We developed the exercise regime for this feasibility study based on the most effective and easiest to implement interventions identified in our systematic review and meta-analysis of primary-care frailty intervention RCTs or cohort studies with control groups.^9^^,^^10^

**Figure 1:**
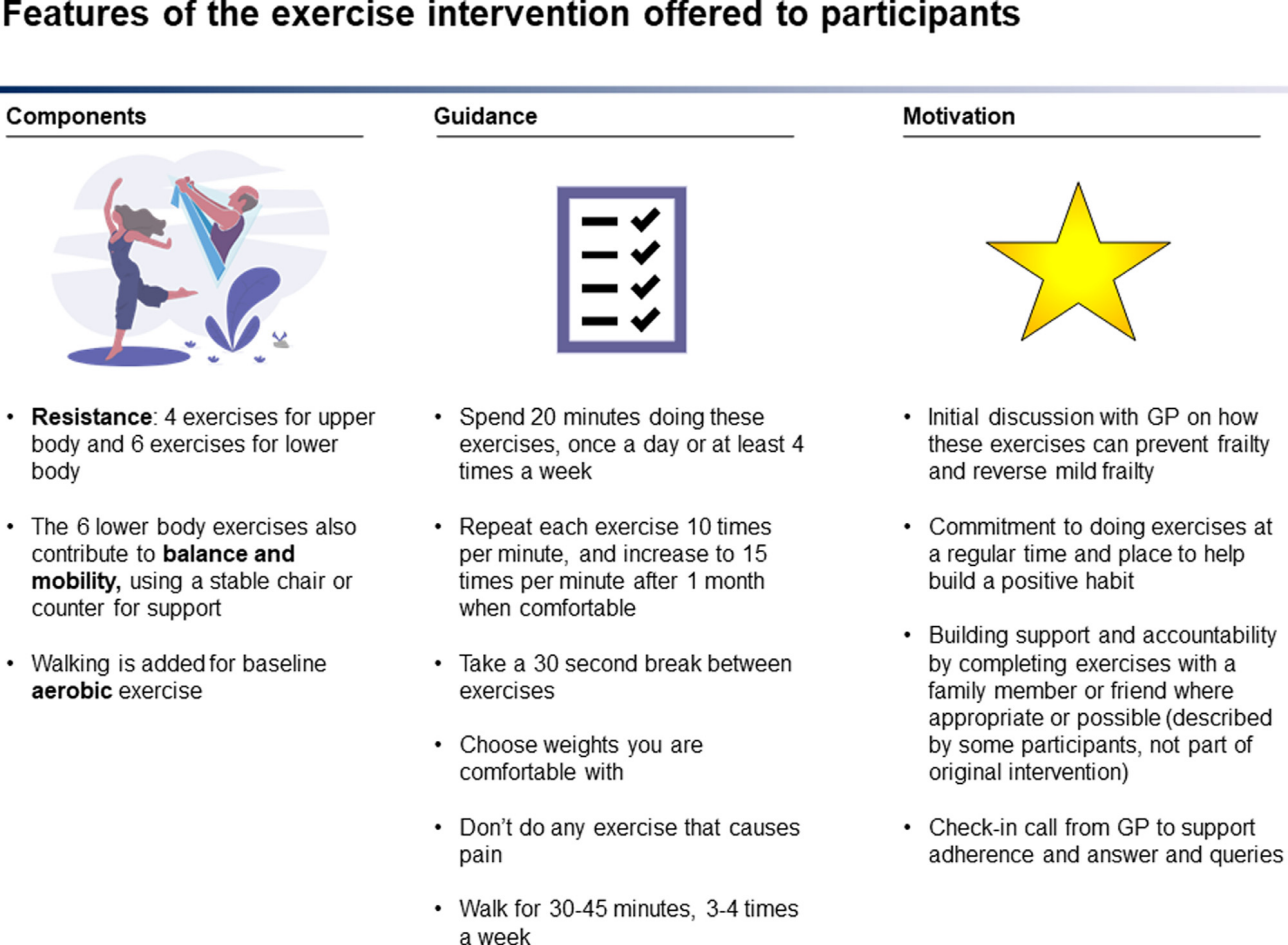
Exercise intervention key features.

An exercise regime appropriate for pre and mildly frail older people was co-designed in two group discussions with eighteen community-dwelling adults, aged 65 and over, attending a local weekly health education programme on aspects of ageing^20^ and invited to participate in the co-design by the principal investigator (PI).^21^ The two-hour long discussions were facilitated by the PI using the Socratic education method.^22^ We discussed key aspects of frailty in the first meeting, namely: definition, risks, screening and interventions and sought feedback on the demand for and practicality of an exercise intervention. A draft exercise regime based on feedback was provided and demonstrated in the second meeting and additional feedback sought. The intervention was further refined with multi-disciplinary team input from physiotherapy, gerontology, and GP colleagues (key features shown in Figure 1).

The feasibility of this co-designed exercise intervention was then tested with new participants. Every older adult presenting to a primary-care practice over a six-month period from January 2020, meeting eligibility criteria, was invited by their GP to participate in the feasibility assessment, with informed consent. Inclusion criteria were adults aged 65 and over with a score of 5 (mildly frail) or less on the clinical frailty scale (CFS).^23^ Exclusion criteria were persons under 65 years old; moderately or severely frail on the CFS; presenting with need for emergency care; persons in residential care; diagnosed with dementia; or needing to be accompanied by a support person.

They were offered the co-designed pictorial guide with resistance-based exercises and an educational discussion by their GP. The GP defined frailty and resilience, described how resistance exercises can strengthen muscles and bones to prevent or reverse frailty, and demonstrated the exercises. Demographic information, vital signs, BMI, handgrip strength, and multi-morbidities, were recorded. CFS score was assigned at enrolment and phenotypic frailty status was quantified using the SHARE Frailty Instrument (SHARE-FI) for primary care. SHARE-FI is a validated, gender-specific tool based on exhaustion, weight loss, handgrip strength, slowness and low activity.^24^

One-on-one telephone interviews were conducted with half the participants at one month and all participants at two months. Patients were randomised on a 1:1 basis to receive the one month call to determine how that additional follow up contact might affect subsequent adherence. Characteristics of each group were analysed for differences at baseline. Means and medians were reported as appropriate for continuous measurements, with frequencies and percentages for categorical measurements. Baseline differences were tested using either independent *t*-test or Mann-Whitney *U test* for continuous measurements, depending on measurements being normally distributed or not, while a chi-squared test was applied to determine potential differences in categorical measurements (using IBM SPSS version 26). Statistical analysis of the adherence outcome was done by binomial regression analysis and interpreted using a 5% level of significance.

Participants were asked by phone if they had followed the exercise regime, and if so, for how long and how many times a week. Ease of doing the exercise regime was recorded on a five-point Likert scale by asking if they found it ‘very easy’, ‘somewhat easy’, ‘neither easy nor hard’, ‘somewhat hard’, or ‘very hard’. Self-reported difference to general health as a result of doing the exercises was recorded on a five-point Likert scale by asking if they felt ‘much better’, ‘slightly better’, ‘about the same’, ‘slightly worse’ or ‘much worse’.

Chi-squared tests followed by binomial tests were used to determine the difference in proportions across response types to ease of doing the exercises and general health as a result of the exercises. Multiple comparisons of responses to the same question were controlled for using the Bonferroni correction method. All statistical testing was interpreted using a 5% level of significance.

Lastly, participants were asked an open question if they had any feedback or would change the exercise regime. The PI conducted these calls. Data was recorded in pseudo-anonymous format by the PI in Microsoft Excel and key themes were identified by Framework analysis.^25^

Feasibility was assessed using the Bowen feasibility model.^17^ This approach has been applied in assessing public health and preventive medicine interventions and assesses feasibility across eight areas of focus. We applied the model as follows:

• Acceptability: How potential participants and GPs react to the intervention, including participation rate and reported ease of the intervention.
• Demand: Levels of adherence with the intervention.
• Implementation: The ease with which the intervention can be implemented, including human resource requirements, time, and cost.
• Practicality: Challenges to the intervention being offered in a primary-care setting where there are constraints on time and commitment.
• Adaptation: The need to modify the intervention to meet the needs of participants or the primary-care setting.
• Integration: The need to make system changes (e.g., at a practice level) to integrate an intervention into an existing system.
• Expansion: The ability to apply the intervention designed in a research environment to a primary-care setting.
• Limited efficacy: Testing the intervention in a convenience sample with short follow-up (i.e., two months), including self-reported benefits to health.

The following steps were taken to verify data:

• Data was checked twice by the investigator when entering data into Microsoft Excel to help avoid typographical errors.
• The investigator checked 'out of range' or anomalous data and monitored for any data entry alerts made by the Microsoft Excel software.
• Data was not transcribed manually to another format to avoid transcription errors.
• Co-authors had access to and verified study data.

Ethical approval was granted by the Irish College of General Practitioners research ethics committee on November 9th, 2019. Consent was obtained from all the participants.

### Role of the funding source

No role was played by the source of grant funding for Roman Romero-Ortuno (Science Foundation Ireland) in this study, which includes no role in study design, data collection or analysis, no access to data and no input on writing or decision to submit for publication.

## Results

A total of 94 of 107 eligible people (88%) agreed to participate in the exercise regime. The average age was 77 and 59 were women (63%). 95% of women and 78% of men enroled. Only 15% had previously considered resistance exercises. One participant dropped out due to hospital admission for surgery, unrelated to the intervention. Measured by SHARE-FI, 12 (15%) participants were frail, 26 (33%) were pre-frail, and 42 (52%) were non-frail. CFS scores were in the range 2 to 5 with a median score of 3 (i.e., people whose medical problems were controlled but were not regularly active). Median CFS score for those who declined to participate was also 3. Participants measured as frail by SHARE-FI had a CFS score no more than 5 (no more than mildly frail). Baseline characteristics are shown in Table 1 and are in line with national or comparable international population statistics for co-morbidities,^26^ grip-strength,^27^ overweight,^28^ activity levels,^29^ and frailty.^30^^,^^31^ Baseline characteristics differed from the wider population in terms of gender^32^: 63% of our sample were women versus 52% nationally, and obesity,^28^ where our sample had a lower prevalence (23% versus 35% nationally).

**Table 1:** Participant baseline characteristics.

	Mean	(SD)	Number	(%)
Age in years	76.4	7.3		
Female			59	62.8
Male			35	37.2
Number of co-morbidities	3.1	1.1		
Hypertension			48	52.2
Ischaemic heart disease			17	18.5
Atrial fibrillation			11	12.0
Myocardial infarction			7	7.6
Stroke			5	5.4
Diabetes			12	13.0
Osteoporosis			12	13.0
Chronic respiratory disease			10	10.9
BMI	26.9	4.6		
BMI 25–29.9			39	41.5
BMI 30–39.9			22	23.4
BMI > 40			1	1.1
SHARE-FI frailty criteria				
Grip strengh female (kg)	20.9	4.4		
Grip strength male (kg)	32.5	7.7		
Exhaustion			26	27.7
Reduced appetite			15	16.0
Slowness			21	22.3
Activity 1 (>once/week)			59	62.8
Activity 2 (once/ week)			18	19.1
Activity 3 (1–3/month)			10	10.6
Activity 4 (hardly ever)			7	7.4
Non-frail			42	52.5
Pre-frail			26	32.5
Frail			12	15.0
Current physical exercise (at least weekly)				
Walking			60	63.8
Golf			11	11.7
Dancing			9	9.6
Swimming			8	8.5
Cycling			3	3.2
Other (e.g., yoga, gardening, gym)			26	27.7

Sheehan, A. and O'Sullivan, R., (2020) Ageing and Public Health – an overview of key statistics in Ireland and Northern Ireland. Dublin: Institute of Public Health.

**Figure 2:**
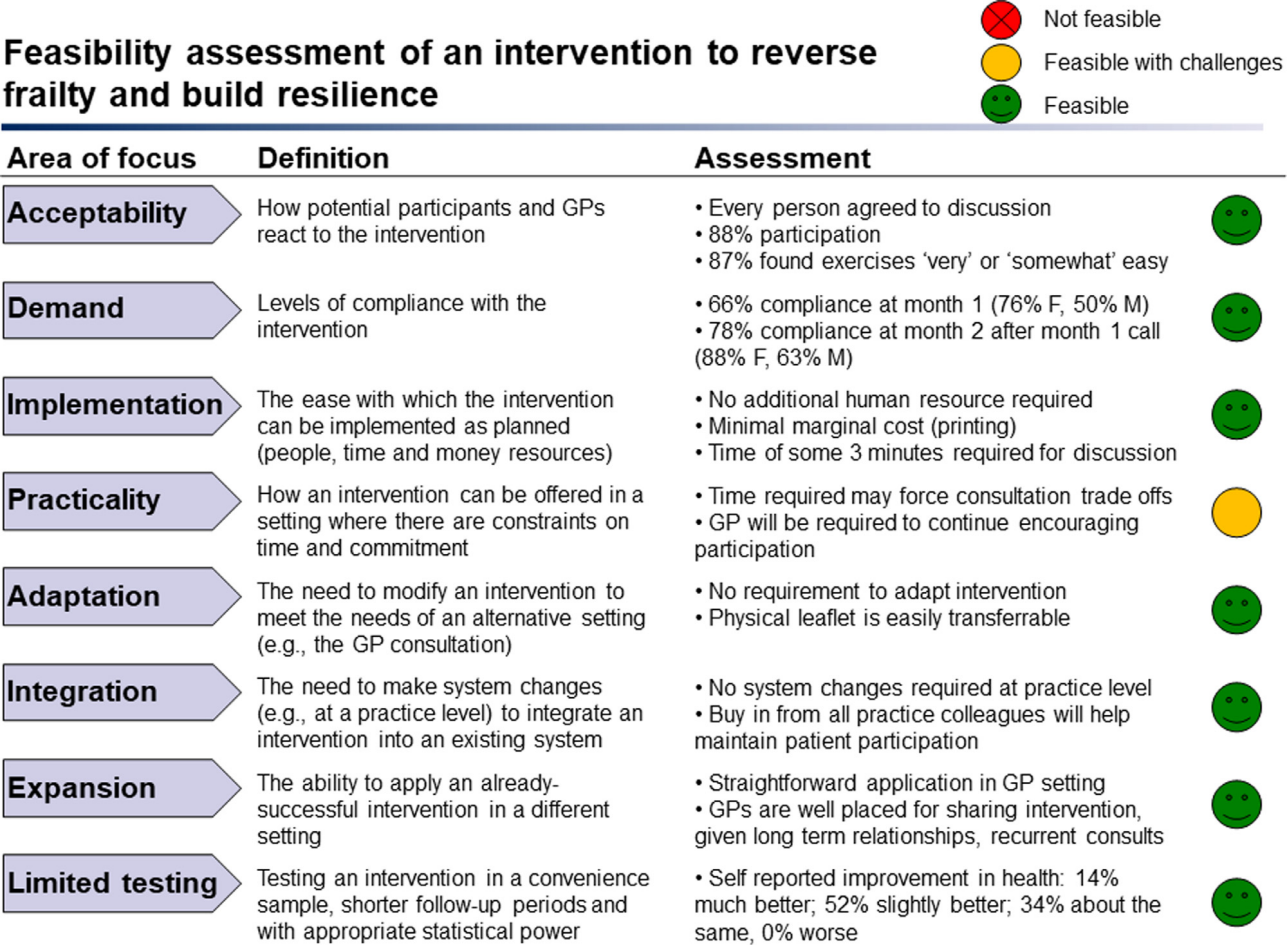
Feasibility assessment overview.

The eight areas of feasibility focus are described below and summarised in Figure 2.

### Acceptability

**Figure 3:**
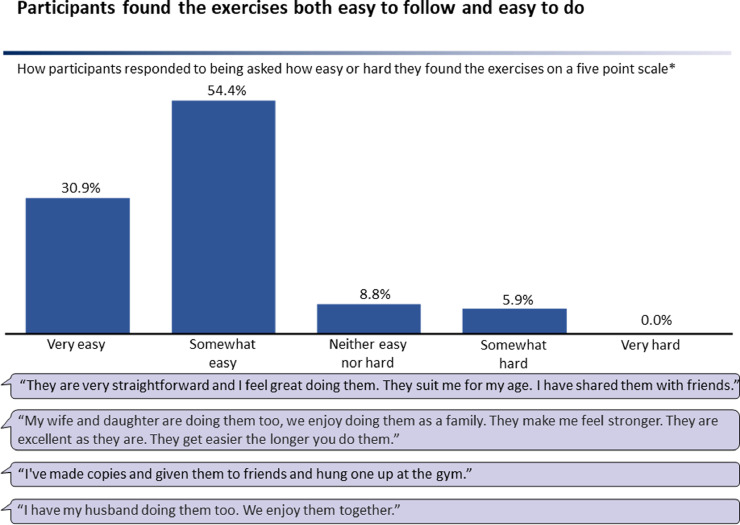
Participant feedback on ease of intervention.

There was a positive reaction from potential participants to discussion on frailty and the offer of an intervention, with no differences noted amongst participations of different frailty states. Every person agreed to a discussion. Intervention participation was 88%. Participants found the exercises both easy to follow and generally easy to do. 31% described exercises as ‘very easy’, 54% ‘somewhat easy’, 9% ‘neither easy nor hard’, 6% ‘somewhat hard, and 0% ‘very hard’ after two months (Figure 3). A significant difference in how participants rated the exercises was found overall (*P* <0.001). There was a statistically equal distribution of participants rating the exercises ‘very easy’ and ‘somewhat easy’ (*P* > 0.05). Ratings in each of these two categories were significantly different compared to each of ‘neither easy nor hard’. ‘somewhat hard’ and ‘very hard’ (*P* < 0.01).

Selected quotes from participants during the one- and two-month calls include:

“They are very straightforward and I feel great doing them. They suit me for my age. I have shared them with friends.”

“We enjoy doing them as a family. They make me feel stronger. They are excellent as they are. They get easier the longer you do them.”

Many described how the home-based exercises helped with staying active and reducing anxiety while housebound during the Covid-19 pandemic:

“The exercises motivate me, they are good for me, they bring discipline and routine into my life especially during (Covid-19) isolation.”

“They are so helpful as we can't get out at all. My training has been cancelled so this is all I have for exercise.”

“I'm stuck inside and I do them almost every single day. They're a gift during the lock-down.”

### Demand

At one month, 65% of participants called were exercising regularly (75% of women, 50% of men). At two months, adherence amongst those who had not been called at one month was: 65% (67% of women, 62% of men). However, adherence amongst those previously called was higher: 78% (88% of women, 63% of men).

There were no significant differences in baseline characteristics between the ‘called’ and ‘not called group’ except for slowness (12 participants self-reported slowness in the ‘called’ group compared to 8 in the ‘not called’ group) (Table 2).

**Table 2:** Baseline characteristics for groups randomised to 'not called' or 'called' at one month.

	Not called at one month (*n* = 46)			Called at one month (*n* = 45)			P
	Mean (SD)	Median	Number (%)	Mean (SD)	Median	Number (%)	
Age in years	75.8 (7.5)	75.0		76.7 (6.6)	77.0		0.467^c^
Female gender, yes			29 (61.7)			28 (62.2)	0.935^a^
Number co-morbidities	3.1 (1.0)			3.1 (1.2)			0.471^b^
BMI	25.9 (4.4)	26.0		27.5 (4.0)	27.7		0.091^c^
Grip strength (kg)	24.6 (7.4)	23.2		26.2 (8.7)	22.9		0.660^c^
Exhaustion			11 (23.9)			15 (33.3)	0.320^a^
Reduced appetite			6 (13.0)			9 (20.0)	0.144^a^
Slowness			8 (17.4)			12 (26.7)	0.037^a^
Activity 1 (>once/week)			30 (65.2)			28 (62.2)	0.935^a^
Activity 2 (once/ week)			8 (17.4)			10 (22.2)	0.958^a^
Activity 3 (1–3/month)			6 (13.0)			2 (4.4)	0.479^a^
Activity 4 (hardly ever)			2 (4.3)			5 (11.1)	0.440^a^
SHARE-FI score	0.5 (1.2)	0.4		1.1 (1.8)	0.5		0.233^c^
Clinical frailty scale	3.0 (0.9)	3.0		3.1 (0.8)	3.0		0.895^a^

a Chi-squared test.

b independent *t*-test.

c Mann Whitney U test.

**Figure 4:**
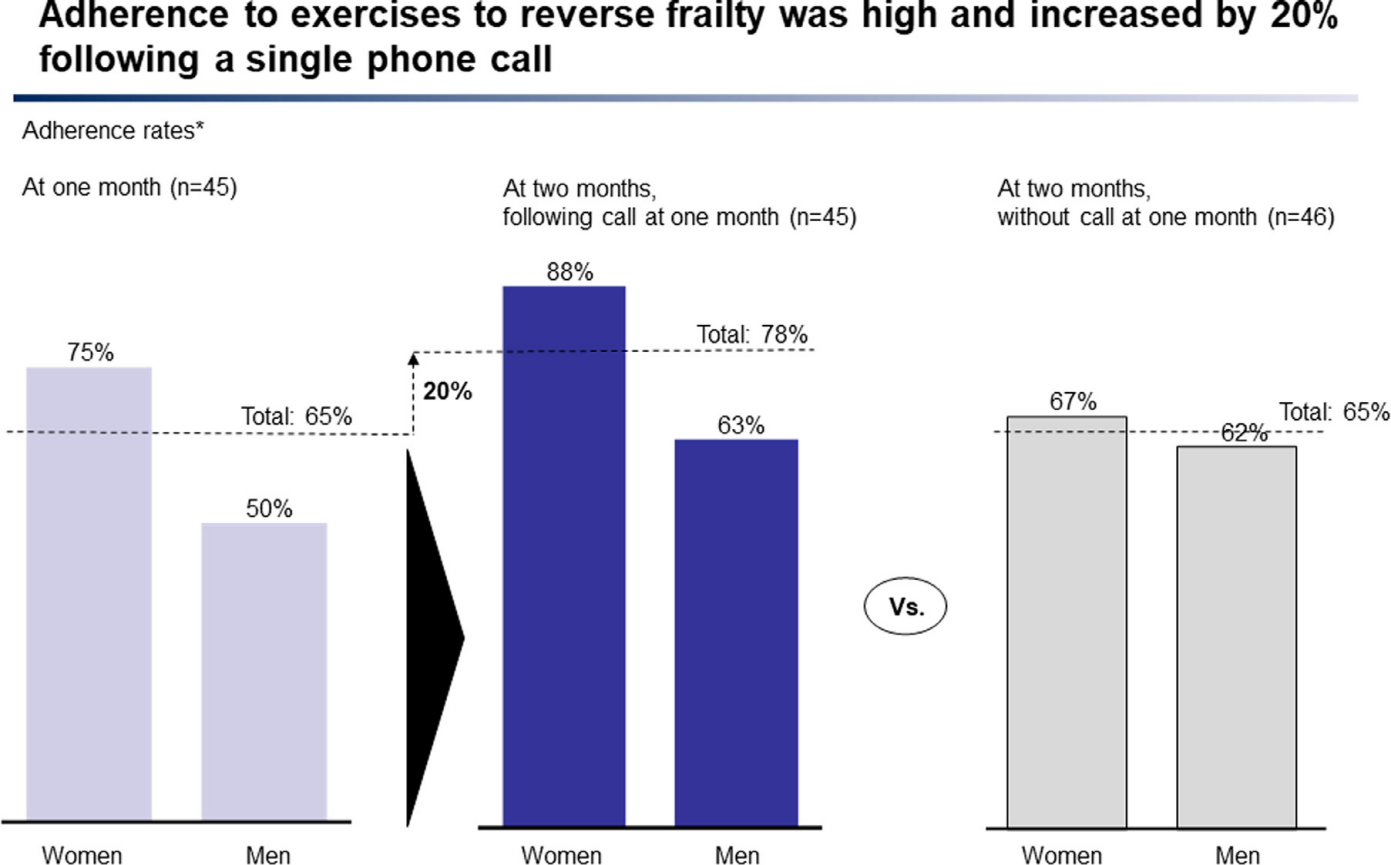
Effect of a single telephone call on adherence.

Binomial logistic regression analysis, adjusted for slowness, showed a statistically significant difference in adherence rates at two months (*P* = 0.017) for those called at one month (*n* = 45) compared to those not called at one month (*n* = 46). The difference was significant (*P* = 0.039) for women called at one month (*n* = 28) compared to women not called (*n* = 29), but not (*P* = 0.420) for men called (*n* = 17) compared to not called (*n* = 17), though the latter may be affected by small sample size. 64% of those who were not doing exercises at one month had taken them up following a single phone call (67% of women, 63% of men) (Figure 4).

### Implementation

No additional primary-care human resources were required to implement the intervention. The marginal cost was small and involved third party printing costs of some 0.05 euro per intervention at scale (commercial printing of the guide on colour, double sided, 170 g quality paper). Time spent introducing and sharing the intervention, excluding time spent on providing a patient information leaflet for the study, gaining informed consent, and gathering data that applied to the feasibility study, was in the range of three to five minutes.

### Practicality

Though the time required for sharing the intervention might appear short, it may force trade-offs in what a GP can cover in a typical ten to fifteen-minute consultation. A commitment to continue encouraging participation in the intervention places additional, ongoing time constraint on the GP in future consultations.

### Adaptation

There were no requirements to adapt the intervention for the purposes of sharing in a primary-care setting. Some feedback underlined the opportunity to adapt the intervention to meet participant preferences. Two participants commented that they would prefer photos of both men and women demonstrating the exercises. Two said they would like to follow a video of the exercises, though the consensus was that a physical sheet was essential and preferred.

### Integration

No system changes were required to integrate the new intervention into the existing infrastructure and processes of the primary-care practice.

### Expansion

Transfer of the intervention from a research environment to a real-world primary-care setting was straightforward. Long term relationships and recurrent consultations that are a feature of primary-care underline the appropriateness of GPs offering this intervention.

### Limited testing

**Figure 5:**
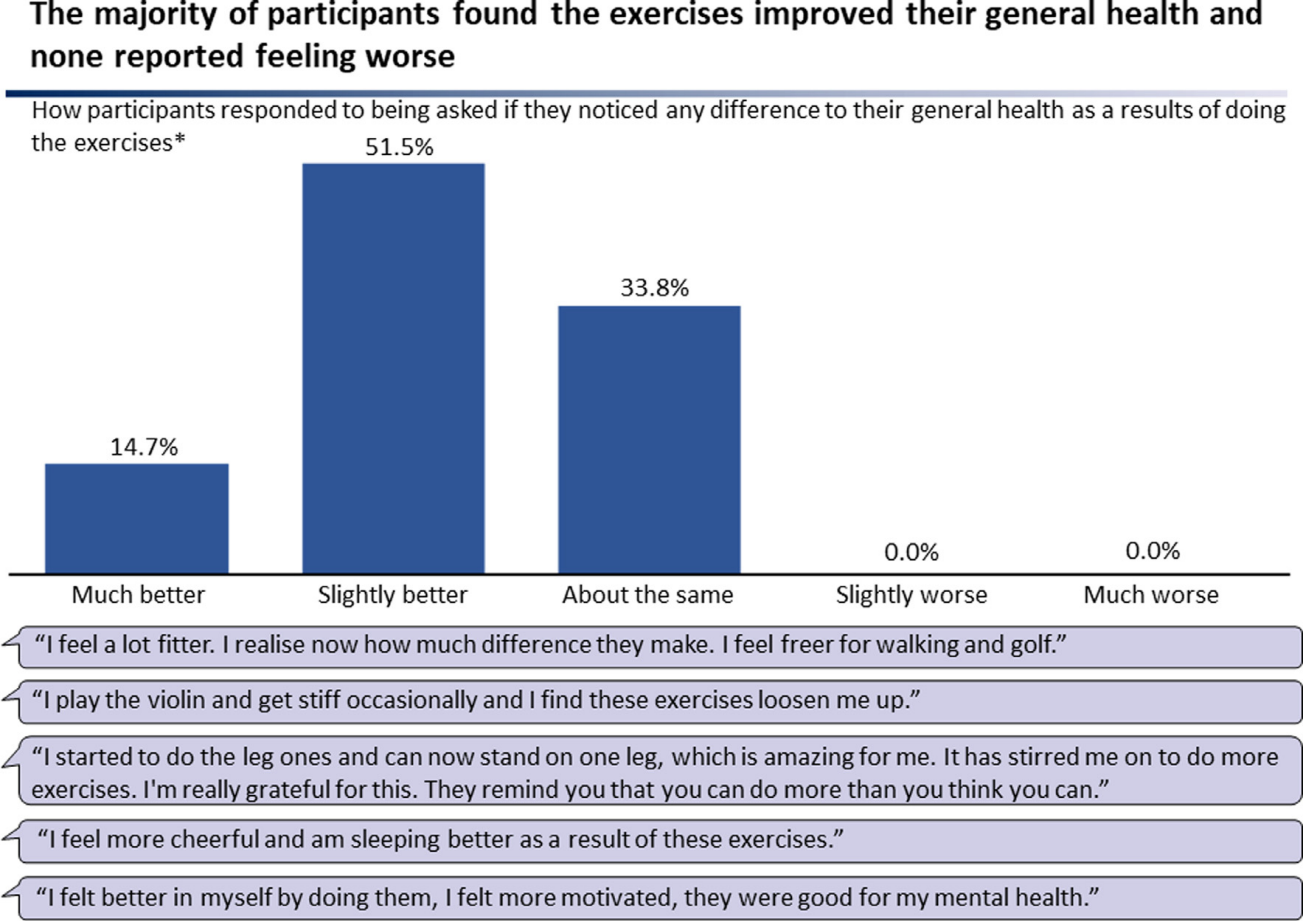
Participant feedback on general health impact.

The majority of participants self-reported their general health had improved as a result of doing the exercises and none reported feeling worse. 14% felt ‘much better’, 51% ‘slightly better’, 34% ‘about the same’, 0% ‘slightly worse’, and 0% ‘much worse’ (Figure 5). There was overall a significant difference in how participants rated impact on general health (*P* < 0.01). In particular, differences between each of ‘slightly worse’ and ‘much worse’ and all three other categories were significant (*P* < 0.01). Many reported mental health benefits. No adverse effects were reported.

Some selected quotes from participants include:

“I feel a lot fitter. I realise now how much difference they make and must keep doing them or will stiffen up. I feel freer for walking and golf.”

“I feel more cheerful and am sleeping better as a result of these exercises.”

“It has stirred me on to do more exercises. I'm really grateful for this. They remind you that you can do more than you think you can.”

An overview of these eight areas of focus is provided in Figure 2.

## Discussion

Our assessment of an optimised frailty intervention in a real-world GP setting has established overall feasibility. There was positive engagement in discussion on frailty and resilience and exercise uptake was high amongst non-frail to mildly frail older adults. Adherence was as high as two-thirds and a single telephone call appeared to help increase participation by 20%. The intervention was feasible for both participants and GPs. The short time required to offer the intervention may still present a challenge in time-constrained consultations. Participants reported meaningful physical and mental health benefits. There were no adverse events reported.

Our assessment bridges a gap between the need for evidence-based tools in a pragmatic setting and the fact that most evidence for behavioural interventions is based on controlled-setting efficacy trials.^18^

Our study suggests strong participant demand for such an exercise intervention with high participation and retention rates.

It was heartening to hear feedback from many participants about how the exercise regime supported mental health, providing a daily routine and means to reduce anxiety during the isolation of national pandemic lockdown.

A limitation arises from excluding participants from our study with a CFS score of 6 or more (i.e., moderately frail to terminally ill). The CFS descriptor for ‘moderately frail’ includes people who ‘need help with all outside activities…often have problems with stairs and need help with bathing’. This co-designed intervention, comprising 10–15 repetitions per minute and 30–45 min walking 3–4 times a week was more appropriate for those with less than moderate frailty. Moderately frail people could benefit from a similar programme assessed in a further study with design and intervention that takes account of their differing needs. Notwithstanding, ‘mildly frail’ remains a key frailty category, where health benefits from this intervention can be achieved. This study addresses an identified gap in evidence for pre-frail and mildly frail older people.^8^

Two frailty tools were applied in this study. While CFS is an appropriate and feasible tool for initial frailty assessment and eligibility screening in primary care, SHARE-FI is based on the physical phenotype approach to the definition of frailty, which considers frailty as a state engendered by comorbidity but as a precursor of disability.^33^ Therefore it underlines a primary care goal to preserve independence and prevent disability. There is growing consensus in academic and clinical communities that applying different frailty tools can be complementary and synergistic.^34^, ^35^, ^36^

The testing of efficacy was limited to self-reported measures as the emphasis of this study was on feasibility parameters that underpin practical application. Feasibility testing was also limited to a single primary-care centre and GP. Although the centre serves a diverse socio-economic group, this limitation increased the risk for unconscious investigator bias as well as limiting data to one geographic area. Methods that can account for confounding, such as stratification or multivariable models, were not applied. However, we have attempted to reduce the risk of confounding by randomly assigning participants to the check-in call and to mitigate the risk of selection bias by assessing every consecutive patient for eligibility. The extent to which retention and adherence may have been enhanced by participants staying at home more often during the pandemic has not been assessed. These limitations will be addressed in a subsequent definitive, multi-centre RCT measuring objective efficacy outcomes.^19^

Although the baseline characteristics were comparable with population statistics for several key measures, our sample had a higher proportion of women and a lower prevalence of obesity compared to the general population. These differences may affect the generalisability of results. This limitation will be addressed in the RCT statistical analysis. The higher number of women reflects differences in attendance rates amongst older women and men visiting general practice,^37^^,^^38^ notwithstanding that difference declining with age.^38^

Feasibility studies of primary-care exercise interventions to address frailty are limited, despite several full controlled trials.^9^

A primary-care feasibility study of an exercise and dietary intervention to reverse or delay frailty amongst older African-Americans found similarly high participation and satisfaction ratings and met feasibility criteria.^39^ However, the intervention was ‘low-dose’, with just one session per month delivered by an occupational therapist. 65% of participants were women, comparing closely with 63% in our study.

A study testing feasibility and effectiveness of a nurse-led community exercise programme for frail older people in Japan found improved physical function and emotional status.^40^ High participation was similar to our study. However, primary focus of this trial was on efficacy measures, with little attention to the seven other feasibility parameters, such as acceptability or practicality.

A feasibility study of an intervention to prepare older patients for surgery focussed on a comprehensive geriatric nursing approach to prevent delirium, depression, pressure ulcers and infections, though with no exercise component.^41^

The protocol for a planned feasibility study of a resistance training intervention in residential care settings proposes using the eight Bowen feasibility criteria with similar patient-centred efficacy measures to our study.^42^ It also aims to inform a future clinical trial.

We could identify no feasibility study in the literature that tested a primary-care, GP led, exercise intervention to address frailty in the community.

The findings support the opportunity to offer a straightforward and minimal cost intervention to prevent or reverse mild frailty. They help support key aspects of the bio-psycho-social model of primary-care in the following ways: to improve health outcomes for amongst the most vulnerable people; apply tools that enable care for the whole person, rather than single system conditions; and consider the biological age of patients rather than chronological age.

Significant impact on adherence was achieved with a low cost, short and relationship-building phone call and may reflect the trust based relationship between clinician and patient. A difference in self-reported slowness between the baseline groups of those ‘called’ and ‘not called’ was identified as a possible confounding factor. No clear inference can be drawn from the difference due to the small numbers involved. Analysis was adjusted for slowness to avoid possible confounding.

While overall feasibility criteria were satisfied, the practicality of spending even a few minutes introducing and offering a frailty intervention may force GPs to consider trade-offs in time constrained consultations. They will have to weigh up the potential for significant benefits from such an intervention with the opportunity cost for other patient care. A solution may rest with health care authorities resourcing dedicated GP frailty screening and intervention, similar to existing systems for chronic disease management in the community.

It is notable that women on average demonstrated greater willingness to try the exercises (95% vs 76%) and continued to do them more than men. Older men also lag in frequency of social interaction,^43^ which has also been shown to be a risk factor for physical frailty.^44^ GPs may wish to be mindful of these issues when encouraging male participation.

Separate studies are warranted to assess the benefits of similar interventions for moderately and severely frail people. Additional research could also inform how bespoke interventions for frailty and co-existing chronic conditions, such as diabetes,^45^ could be developed.

The positive results of this study suggest that testing this optimised intervention in a full-scale trial is justified. It is hoped these results can encourage mainstream adoption of feasible interventions to reverse frailty and build resilience in primary-care.

Feasibility criteria for an exercise intervention to prevent or reverse mild frailty in primary-care have been satisfied. Intervention uptake and adherence were high. A single telephone call appeared to help increase adherence by 20%. Participants reported meaningful physical and mental health benefits. The findings informed the design of a definitive RCT.

## Contributors

John Travers: Study conceptualisation and design, literature search, data curation, data collection and analysis, data verification, investigation, methodology, writing of original draft.

Roman Romero-Ortuno: Study conceptualisation and design, methodology, data verification, writing review and editing.

Marie-Therese Cooney: Study conceptualisation and design, methodology, validation, supervision, writing review and editing.

All co-authors had access to all study data and accept responsibility to submit for publication.

### Data sharing

Anonymized feasibility study data is available on request from the corresponding author. A model consent form is available at the Harvard Dataverse repository: https://doi.org/10.7910/DVN/RKEGIV

### Funding

Roman Romero-Ortuno is funded by a grant from Science Foundation Ireland (SFI), grant number 18/FRL/6188

## Declaration of interests

None.
